# Differential Gene Expression in Ovaries of Qira Black Sheep and Hetian Sheep Using RNA-Seq Technique

**DOI:** 10.1371/journal.pone.0120170

**Published:** 2015-03-19

**Authors:** Han Ying Chen, Hong Shen, Bin Jia, Yong Sheng Zhang, Xu Hai Wang, Xian Cun Zeng

**Affiliations:** 1 School of Pharmacy, Shihezi University, Shihezi, Xinjiang, China; 2 College of Animal Science and Technology, Shihezi University, Shihezi, Xinjiang, China; University of Sydney, AUSTRALIA

## Abstract

The Qira black sheep and the Hetian sheep are two local breeds in the Northwest of China, which are characterized by high-fecundity and low-fecundity breed respectively. The elucidation of mRNA expression profiles in the ovaries among different sheep breeds representing fecundity extremes will helpful for identification and utilization of major prolificacy genes in sheep. In the present study, we performed RNA-seq technology to compare the difference in ovarian mRNA expression profiles between Qira black sheep and Hetian sheep. From the Qira black sheep and the Hetian sheep libraries, we obtained a total of 11,747,582 and 11,879,968 sequencing reads, respectively. After aligning to the reference sequences, the two libraries included 16,763 and 16,814 genes respectively. A total of 1,252 genes were significantly differentially expressed at Hetian sheep compared with Qira black sheep. Eight differentially expressed genes were randomly selected for validation by real-time RT-PCR. This study provides a basic data for future research of the sheep reproduction.

## Introduction

Sheep are one of the important domestic animals in Xinjiang province in the northwest of China. High reproductive efficiency is one of the important goals in sheep breeding, because it plays an important role in efficiency of production. However, low-fecundity in the majority of domestic sheep is one of limiting factors for the development of sheep industry. Because of low heritability of the reproduction traits and sex-limited nature, traditional selection has been employed for improving litter size with only limited advancement [1]. So scientists pay more attention to search the candidate genes associated with ovulation rate and multiplets, which can lead to genetic improvement through the implementation of gene and/or marker assisted selection (MAS) [2].

With the development of molecular, sequencing and bioinformatics analysis technologies, high throughput RNA deep sequencing (RNA-seq) provides a platform for measuring large-scale gene expression patterns [3–5]. Recently, in order to identify the differentially expressed genes (DEGs) and novel transcript units, the RNA-seq has been widely used in domestic animals, including goats [6–7], pigs [8], cows [9], sheep [10–12], and others. Additionally, the efficacy of RNA-seq has also been demonstrated in mammalian reproductive tissues, for example in pig gonad [8, 13–14], bovine blastocyst [15–16], bovine granulosa cell [17], goat ovary [19], sheep oocyte and granulosa cell [18], and sheep ovary [20]. However, reports on the sheep DEGs and transcriptome information related to litter size or prolificacy trait that have applied RNA-seq technology are very limited. One previous study with the use of RNA-seq technology in ovary tissues reported that 1,924 and 1,501 DEGs were identified between the Surabaya fur sheep with the *FecB* (Booroola gene) genotype BB Han sheep and *FecB* genotype ++ Han sheep respectively [20].

Qira black sheep and Hetian sheep are two local breeds in Hetian region, Xinjiang province, China. The former is characterized by year-around estrous behavior and high-fecundity with an average lambing rate of 215.5% [21], while the latter is a low-fecundity breed with an average lambing rate of 102.5% [22]. The difference in fecundity between Qira black sheep and Hetian sheep may be associated with the difference of ovulation rate [23]. Significant fecundity differences between Qira black sheep and Hetian sheep have provided appropriate animal models for the identification and utilization of sheep major prolificacy genes [20]. In the present study, we detected the differential expression profiling in ovaries of the mRNAs in two groups (Qira black sheep and Hetian sheep) using RNA-seq technology. This work advances our understanding of the molecular mechanism of sheep fecundity, and provides basic data for future studies.

## Materials and Methods

### Ethics statement

The sheep experiments were performed in accordance with the “Guidelines for Experimental Animals” of the Ministry of Science and Technology (Beijing, China) and the “Regulations for the Experimental Animal Use Management” of Shihezi University. All ewes were housed in one group under the normal condition of natural lighting and free access to feed. All ewes were slaughtered humanely by anesthesia (Xylidinothiazoline, 1 mg/Kg; Feilong animal pharmaceutical factory, China), and performed by the professional workers at Qira Cattle and Sheep Abattoir.

### Animal and ovary collection

According to the reproduction records, age and body size, Qira black ewes (high-fecundity individuals) and Hetian ewes (low-fecundity individuals) of similar age (3–4 years old) and body size were obtained from the Qira Sheep Breeding Farm located in Qira County, Hetian region, Xinjiang Province, China. A teaser ram was used to monitor the occurrence of estrus for all ewes twice a day. Four Qira black ewes and five Hetian ewes were killed between 12 and 24 h after spontaneous estrus was detected. Ovaries were dissected and all samples were collected with better ovulation points or preovulatory follicles on the surfaces of the ovaries. All samples were immediately frozen on liquid nitrogen for total RNA extraction.

### RNA isolation and RNA-seq

Trizol reagent (Invitrogen, Carlsbad, CA, USA) was used to extract the total RNA from ovaries in the two groups following the manufacturer’s instructions, and then the DNase I was used to degrade any possible DNA contamination. According to the manufacturer’s manual, sequencing libraries were performed at Beijing Genomics Institute (BGI, Shenzhen, China) using the Illumina Truseq RNA Sample Preparation Kit (Illumina, San Diego, USA). Briefly, using the oligo (dT) magnetic beads, the mRNA was enriched from the total RNA and then the purified mRNA was interrupted into short fragments (about 200 bp) by the fragmentation buffer. Random hexamer-primer was added to synthesize the first strand cDNA. Adding buffer, dNTPs, DNA polymerase I (New England Biolabs) and RNase H (Invitrogen), the second strand cDNA was synthesized, and subsequently the double stranded cDNA was purified using the Qiaquick PCR extraction kit, washed with EB buffer for end repaired and Poly (A) tail addition. Finally, after ligating the sequencing adaptors (Illumina PE adaptors), the suitable fragments were selected for PCR amplification according to the results of agarose gel electrophoresis. The cDNA libraries were used for sequencing on an Illumina HiSeq 2000 instrument at BGI—Shenzhen, China.

### Bioinformatics analysis

All clean reads were obtained by rejecting low quality sequence (more than half of the base qualities less than 5) or reads with more than 10% unknown nucleotides (N), and sequencing adapters. The clean reads were aligned to the gene sequences (downloaded from NCBI database) and sheep genome (oar3.1) through SOAP2 [24], with allowing less than 2 mismatches in the alignment. Unmapped or multi-position matched reads were excluded from further analyses. In addition, sequence saturation analyses of the two libraries were executed to provide an overview of the project.

### Expression profiling

The number of mapped reads for each gene was normalized and calculated by using the RPKM (reads per kb per million reads) method, which is an effective method for eliminating the influence of sequencing discrepancy and gene length [25]. Based on the method described by Audic and Claverie [26], we determined the statistical significance of differential expression profile for each gene. The threshold of P-value was determined according to the false discovery rate (FDR) [27]. In this study, to judge the significance of gene expression difference, the absolute value of log_2_ Ratio ≥ 1 and FDR ≤ 0.001 were used as the criteria [28].

### Gene Ontology (GO) and KEGG pathway enrichment analysis

As an international standardized gene functional classification system, GO offers a dynamic-updated controlled vocabulary and a strictly defined concept to comprehensively describe properties of genes and their products [20]. In order to identify the significantly enriched GO terms, all DEGs were mapped to GO terms in GO database (http://www.geneontology.org/) by hypergeometric test [29]. And the Bonferroni-corrected P-value ≤ 0.05 was used as the threshold. GO covers three domains: cellular component, molecular function and biological process [29]. The GO annotation analysis and the plotting of GO annotations were performed using WEGO software [30]. Moreover, according to the KEGG database, the significantly enriched biological pathways were identified [31]. Pathways with a Q-value ≤ 0.05 were considered significant.

### Quantitative real-time PCR (qRT-PCR) confirmation

In order to verify the RNA-seq results, eight candidate genes were selected and detected using qRT-PCR. Three genes (follistatin-related protein 3 precursor, latent-transforming growth factor beta-binding protein 4, and transforming growth factor beta-1-induced transcript 1 protein) were associated with TGFβ; two genes (cytochrome P450 19A1 and steroidogenic acute regulatory protein) were associated with hormone regulation; and three genes (estrogen receptor beta, prolactin receptor, and scavenger receptor class B member 1) were grouped as receptor. β-actin was used as a reference control. Total RNA (1.0 mg) was used to synthesize first-strand cDNA using EasyScript RT reagent Kit (Transgen, Beijing, China) and the qRT-PCR was performed using qPCR SuperMix Kit (Transgen, Beijing, China). The amplification conditions were 95°C for 2 min of initial stage, followed by 40 cycles of 95°C for 10 s and 60°C for 30 s and performed on Mx3000P System. The reactions of all genes were run on one plate in triplicate for each biological replicate. Values of real-time RT-PCR were calculated by the 2^−ΔΔ*C*t^ method [20, 32]. The relative expression level of each gene (Δ*C*t) was calculated as follows: *C*t _target gene_—*C*t _β-actin_, and the relative expression level of each gene in two sheep (ΔΔ*C*t) was calculated as follows: *C*t _Hetian sheep_—*C*t _Qira black sheep_ [20, 32].

## Results

### Sequencing data summary

**Fig 1:**
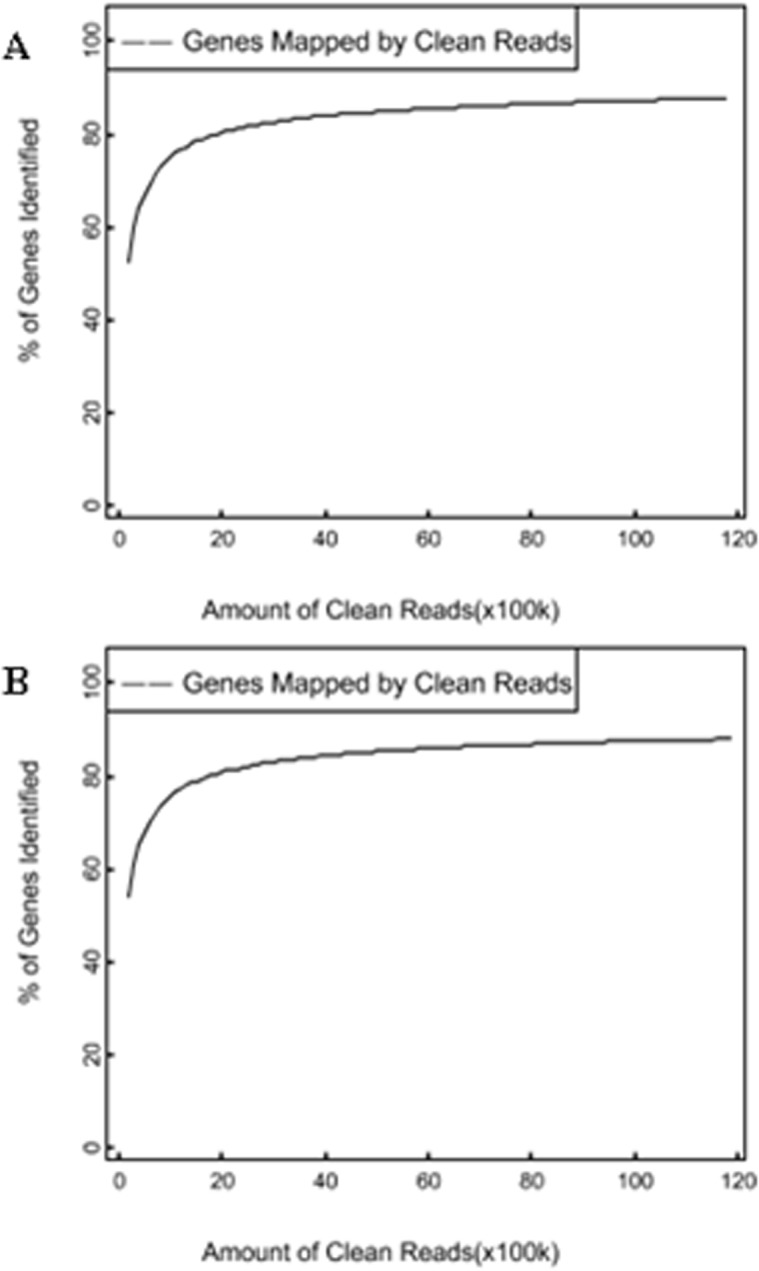
Saturation analyses of Qira black sheep (A) and Hetian sheep (B). The number of detected genes continued increasing as the total number of sequencing reads increased. When the number of reads reaches a certain amount, the number of detected genes almost ceased increasing.

In this study, we sequenced two cDNA libraries from the Qira black sheep and Hetian sheep, respectively, using a Illumina HiSeq 2000 sequencing platform at BGI-Shenzhen, China, and approximately 2.36 Gb reads (1.17 and 1.19 Gb for the Qira black sheep and Hetian sheep, respectively) were obtained. Next, according to the BGI bioinformatics protocols for RNA-seq, these data were preliminarily analyzed. The major characteristics of the two DGE libraries are described in Table 1. The result showed varying amount of sequencing reads for these samples. In both libraries, although 41.99% of the reads in Qira black sheep and 43.81% of the reads in Hetian sheep could be mapped to reference genes, approximately 82% of the reads mapped to sheep genome (Qira black sheep with 82.39%, Hetian sheep with 82.42%). For the unique match, a little more than one-third and two-thirds of the reads corresponded to reference genes and genome respectively. In addition, 33.76% of the reads in Qira black sheep and 34.65% of the reads in Hetian sheep could be perfectly matched to the reference genes, and approximately 65% of the reads perfectly matched to genome. The results of saturation analyses (Fig. 1) showed that when the number of sequenced reads reached 2 M or more, the number of detected genes almost ceased increasing, which validates the integrity of the library for use in further analysis.

**Table 1:** A summary of the sequencing reads alignment to the Ovis aries genome and reference genes.

Sample	alignment to genome		alignment to reference genes	
	Qira black sheep	Hetian sheep	Qira black sheep	Hetian sheep
Total Reads	11,747,582	11,879,968	11,747,582	11,879,968
Total Base-Pairs	575,631,518	582,118,432	575,631,518	582,118,432
Total Mapped Reads	9,679,005(82.39%)	9,790,981(82.42%)	4,932,905(41.99%)	5,204,429(43.81%)
Perfect Match	7,605,875(64.74%)	7,712,860(64.92%)	3,965,493(33.76%)	4,116,347(34.65%)
< = 2bp Mismatch	2,073,130(17.65%)	2,078,121(17.49%)	967,412(8.23%)	1,088,082(9.16%)
Unique Match	8,052,464(68.55%)	8,252,218(69.46%)	4,061,762(34.58%)	4,435,953(37.34%)
Multi-position Match	1,626,541(13.85%)	1,538,763(12.95%)	871,143(7.42%)	768,476(6.47%)
Total Unmapped Reads	2,068,577(17.61%)	2,088,987(17.58%)	6,814,677(58.01%)	6,675,539(56.19%)

### Identification and analysis of DEGs

**Fig 2:**
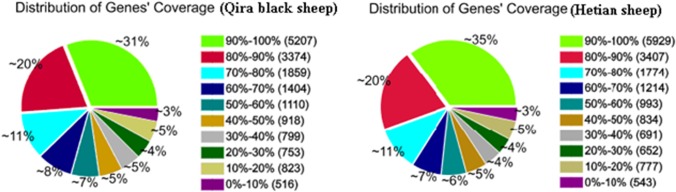
Distribution of genes′ coverage in the two ovine libraries. Gene coverage is the percentage of a gene covered by reads. This value is equal to the ratio of total base count in a gene covered by uniquely mapped reads to the total base count for that gene.

The RPKM method was used to evaluate the gene expression levels. As a result, 16,763 and 16,814 reference genes were identified from Qira black sheep and Hetian sheep libraries, respectively, which shared 16,425 genes in common (S1 Table). As shown in Table 2, approximately 88% and a little more than 1.7% of the reference genes were expressed at less than 100 RPKM and more than 500 RPKM respectively. As shown in Fig. 2, 20% of the reference genes had 80–90% coverage, and 31% and 35% of the annotated genes had 90–100% coverage in Qira black sheep and Hetian sheep libraries respectively, suggesting that the read distributions are similar between the two libraries.

**Table 2:** Gene expression and annotation by RPKM.

RPKM	Gene number of Hetian sheep (%)	Gene number of Qira black sheep (%)
0–50	12,513 (74.42%)	12,538 (74.80%)
50–100	2233 (13.28%)	2243 (13.38%)
100–500	1779 (10.58%)	1685 (10.05%)
>500	289 (1.72%)	297 (1.77%)
Total	16,814	16,763

**Fig 3:**
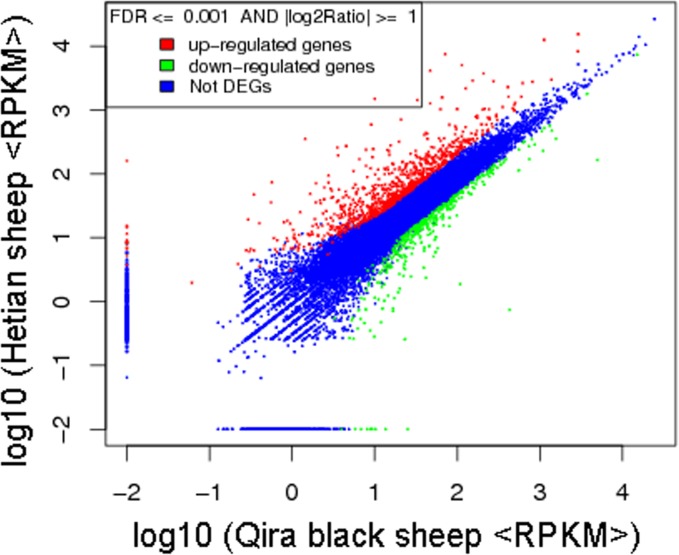
Comparison of gene expression levels between the two libraries. The expression levels are estimated by RPKM value.

To judge the significance of differences in expressed genes, FDR ≤ 0.001 and the absolute value of log2 Ratio ≥ 1 were used as the threshold. A total of 1,252 significantly differentially expressed genes were identified between the two libraries, with 859 genes upregulated and 393 genes downregulated at Hetian sheep compared with Qira black sheep (S2 Table and Fig. 3). Among these genes, 9 genes 13 genes were found only expressed in the Qira black sheep library and Hetian sheep library respectively. The breakdown of fold-change among the DEGs was showed in Table 3. Interestingly, the number of genes having greater than four-fold difference and two-fold difference accounted for 7.03% and 29.79% of the total DEGs respectively.

**Table 3:** Fold change differences in expression of genes differentially expressed between the two sheep at FDR < 0.001.

	Fold change			
	> 6	4–6	2–4	< 2
Genes over-expressed in Hetian sheep	23	42	217	577
Genes over-expressed in Qira black sheep	11	12	68	302

### GO and pathway enrichment analysis

**Fig 4:**
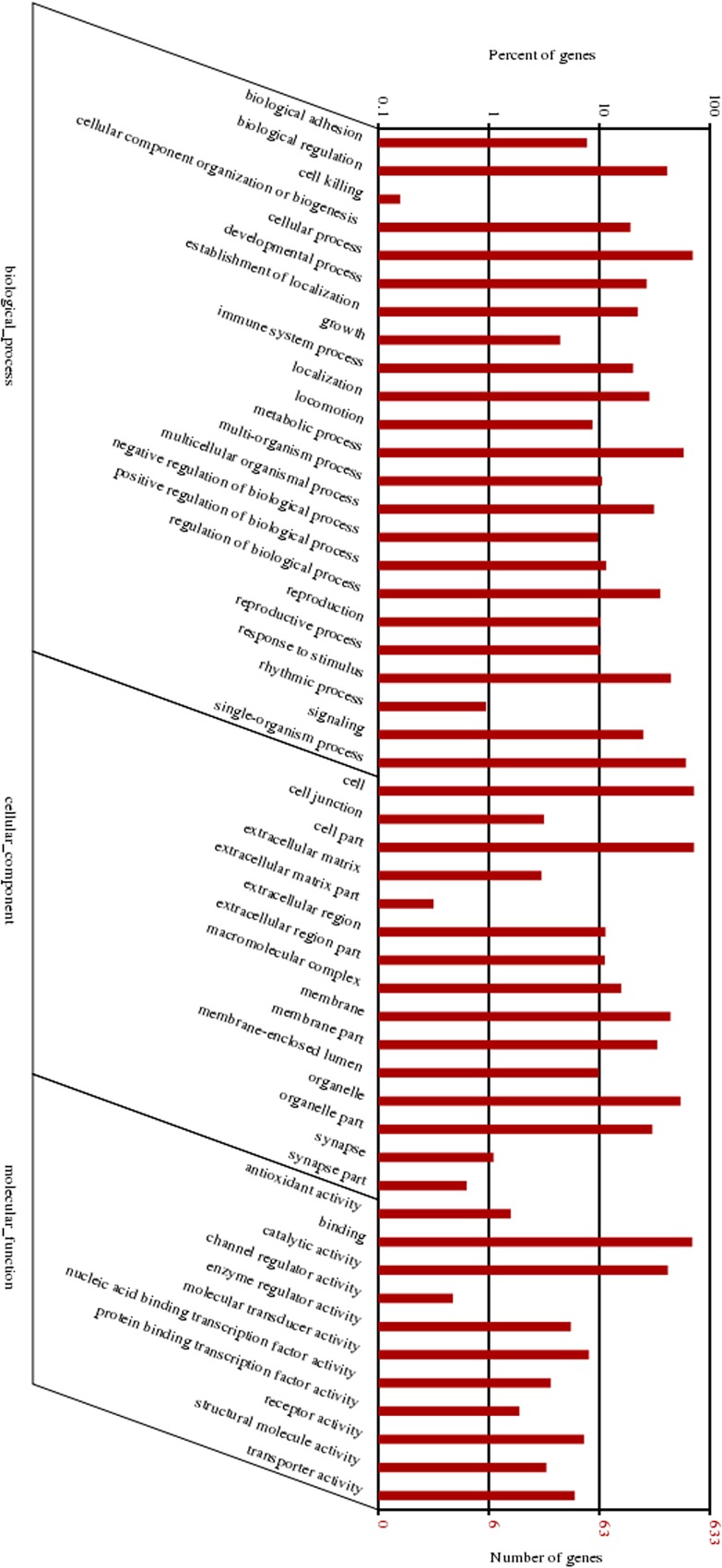
GO analysis of differentially expressed genes between the two breeds. The differentially expressed genes are classified into three categories: cellular component, molecular function, and biological process. The left axis shows the percentage of genes in a category, and the right axis the number of genes.

The enrichment of DEGs in GO terms was tested to gain insights into the biological implications. And several GO terms significantly enriched for DEGs were found and shown in Fig. 4. The GO annotation indicated that the DEGs were involved in many biological processes, such as reproduction, cellular process, metabolic process, biological regulation, developmental process, signaling, growth, and immune function. The main functional groups of DEGs in cellular component are cell, cell part, membrane and organelle, and in molecular function are catalytic activity and binding.

Based on the KEGG pathway database, the pathway analysis was performed to predict the significantly enriched metabolic pathways and signal transduction pathways in DEGs. After pathway enrichment analysis, the significantly enriched pathways were listed in S3 Table. In total, 623 DEGs had KEGG pathway annotations. The results showed that the significant signaling pathways included 218 pathways, for example metabolic pathways, cell adhesion molecules, steroid (hormone) biosynthesis, TGFβ signaling pathway, oxidative phosphorylation, PPAR signaling pathway, and Wnt signaling pathway.

### QRT-PCR confirmation

In the same RNA samples, the expression fold changes (2^−ΔΔ*C*t^) of eight genes were tested by using qRT-PCR to validate the RNA-seq results. And the expression patterns of eight genes were general consistent with the RNA-seq results (Table 4), suggesting that the results of the RNA-seq experiments was efficient and accurate.

**Table 4:** Validation of selected RNA-seq based gene expression by real-time RT-PCR analysis.

Gene	Description	Hetian sheep/Qira black sheep		Primer sequence
		RNA-seq	QRT-PCR	
*CYP19A1*	gi|172088165|ref|NP_001116472.1|/0/cytochrome P450 19A1	3.38	7.89	F: TCGTCCTGGTCACCCTTCTG
				R: CGGTCTCTGGTCTCGTCTGG
*ESR2*	gi|57619192|ref|NP_001009737.1|/0/estrogen receptor beta	−1.58	−0.96	F: TGATGTCCTTGACCAAGCTG
				R: CACTTGGTCGTACAGGCTGA
*FSTL3*	gi|115496578|ref|NP_001069178.1|/6.39084e-111/follistatin-related protein 3 precursor	1.86	1.96	F: GGCTGAATGCTGTGCCTCTG
				R: CCCTCGCACGAATCTTTG
*LTBP4*	gi|426243822|ref|XP_004015745.1|/4.81709e-99/PREDICTED: LOW QUALITY PROTEIN: latent-transforming growth factor beta-binding protein 4	−1.59	−0.84	F: GTCGGTGTTGGTACAGATG
				R: GCAGACGTGAACGAGTG
*PRLR*	gi|21263860|sp|O46561.1|PRLR_SHEEP/0/RecName: Full = Prolactin receptor; Short = PRL-R	5.40	2.94	F: CTTACCACAACATTGCTGACG
				R: GTTTAGCAGAGAACAAGGGG
*SCARB1*	gi|426247178|ref|XP_004017363.1|/5.24272e-135/PREDICTED: scavenger receptor class B member 1	2.09	1.01	F: TGGCCTCCATCCTAACG
				R: TCGAACCACATTAGCGG
*STAR*	gi|57163979|ref|NP_001009243.1|/6.49941e-160/steroidogenic acute regulatory protein, mitochondrial precursor	6.47	2.61	F: TCCATGTGCGTGCTGGCTGG
				R: GGAGCACCATGCAGGTGGGG
*TGFβ1I1*	gi|466003579|ref|XP_004268779.1|/1.70899e-154/PREDICTED: transforming growth factor beta-1-induced transcript 1 protein	−1.42	−0.74	F: CAGGTCTTTCGAGGTGG
				R: CTTCACCTGCACCTTCTG
*β-actin*				F: TCCGCAAAGACCTCTACG
				R: CGGAGTACTTGCGCTCA

Positive and negative values are either up- or downregulated genes in the comparisons. RNA-seq data are shown Log2 ratio. QRT-PCR data were calculated by the 2^−ΔΔ*C*t^method [20, 32] with β-actin as an internal control.

## Discussion

The RNA-seq technology is a powerful approach for identification the expression levels of thousands of genes simultaneously in various tissues including ovary [20]. In this study, differences in gene expression between Qira black sheep and Hetian sheep ovaries were analyzed using RNA-Seq technology. The Qira black sheep and Hetian sheep were the similar age, body size and came from the same region, but their fecundity was very different (the average lambing rate of Qira black sheep was 215.5% and Hetian sheep was 102.5%). The different fecundity may be associated with the different regulation of gene expression, which is involved in the ovulation or follicular development [23]. The results of differential gene expression are more likely to reflect the differences between Qira black sheep and Hetian sheep, and will be valuable for future studies on the identification of major genes or novel genes that affect sheep fecundity.

In this study, A total of 1,252 genes were significantly differentially expressed, some of the DGEs corresponding to genes previously associated with prolificacy processes, such as prolactin receptor (*PRLR*), progesterone receptor (*PGR*), estrogen receptor beta (*ESR2*), cytochrome P450 19A1 (*CYP19A1*), cytochrome P450 11A1 (*CYP11A1*), estradiol 17-beta-dehydrogenase 12 (*HSD17B12*), inhibin beta A (*INHBA*), while other genes may be involved in the follicular development or control of ovulation. For example, we identified members of vascular endothelial growth factor (VEGF) pathway including VEGF receptor 2 (*VEGFR2*) upregulated in Hetian sheep, and VEGF B isoforms (*VEGFB*) upregulated in Qira black sheep. Angiogenesis is critical for the female ovulatory cycle including follicular development, ovulation, and corpora lutea formation [33]. It has been demonstrated that VEGF system constitute the most important signaling pathway in angiogenesis [34], and play an important role in the process of follicular development through the regulation of thecal angiogenesis [34–35]. In particular, several studies reported that *VEGF* can increase rat follicular cell proliferation and inhibit the apoptosis [34], stimulate ovarian follicular development [35–36], and control follicle progression and luteogenesis [37]. Another example is *STAR*, which is involved in the regulation of steroid hormone biosynthesis and follicular development in the mammalian ovary [38–39]. In this study, the expression differences between the Qira black sheep and Hetian sheep ranged from 13.96- to 1.0-fold. The expression differences of 879 DEGs were relatively small (less than 2-fold), but some of them may have large effects on prolificacy processes involved in follicular development, ovulation, litter size, hormone biosynthesis, or others [40].

To investigate the biological functions of the DEGs, we performed the GO annotation and KEGG pathway analysis. This study clearly revealed that some hormone related genes involved in steroid biosynthesis and steroid hormone biosynthesis pathways toward upregulated in Hetian sheep. For steroid biosynthesis pathway, we found seven genes assigned to almost all steps from lanosterol 14-alpha demethylase-like to lathosterol oxidase. Eight of nine genes (*CYP11A1*, *CYP19A1*, *HSD17B12*, hydroxyl-delta-5-steroid dehydrogenase, and others) involved in the steroid hormone biosynthesis pathway were upregulated in Hetian sheep, and one gene (estradiol 17-beta-dehydrogenase 1) was upregulated in Qira black sheep. These genes might be an indirect or direct effect on steroid hormone biosynthesis, and their expression profiles in this study were consistent with the expression profiles of *SCARB1*, low-density lipoprotein receptor (*LDLR*), very low-density lipoprotein receptor isoform 2 (*VLDLR*) and *STAR*. *SCARB1*, *LDLR*, and *VLDLR* have been shown to be involved in biosynthesis of steroid hormones through effects on the absorption of cholesterol substrates from circulating lipoproteins [41–43]. And *STAR* has been shown to be involved in the transportation of intracellular cholesterol from the outer mitochondrial membrane to the inner mitochondrial membrane, thereby regulating the biosynthesis of steroid hormones [42–43]. Other genes related to steroid hormones and upregulated in Qira black sheep are *ESR2* and *PGR*, which are considered important ovarian factors in regulation of female reproduction [3].

As the important intraovarian growth factors, some members of the transforming growth factor-beta (TGFβ) superfamily have emerged as essential regulators of sheep ovarian follicles growth, differentiation and ovulation [44]. Follistatin-related protein 3 is a circulating glycoprotein, and acts as an endogenous inhibitor of TGFβ ligands such as bone morphogenetic proteins and activin [45–46]. Numerous studies have shown the roles of activins in many reproductive processes, such as activins has been shown to promote ovine preantral follicle and oocyte growth in vitro [47], stimulate the rodent ovarian follicular development, and inhibit oocyte apoptosis [48]. One study has shown the crucial roles of follistatin-related protein 3 in limiting testis organ size and promoting age-related testicular regression [49]. We identified *FSTL3* upregulated in Hetian sheep, which implied that *FSTL3* may play a negative effect on fertility in Hetian sheep by regulating activin and members of the BMP subfamily [50].

The RNA-seq and qRT-PCR results showed that several members of TGFβ associated genes were upregulated in Qira black sheep, implied that TGFβ played important roles in high prolificacy of Qira black sheep. Previous work had suggested that latent TGFβ binding proteins (*LTBPs*) might play an important role in TGFβ activation [51]. Miao et al. (2013) have proved that *LTBP4* gene and *TGFβ1I1* gene were highly upregulated in Small-tail Han sheep (high-fecundity; with *FecB* BB or ++ genotype) compared with Surabaya fur sheep (low-fecundity) [20]. In our study, *LTBP4* gene and *TGFβ1I1* gene were highly upregulated in Qira black sheep, implying that they might play critical roles for high prolificacy. And the results suggested that other members of TGFβ superfamily were also involved in the female fertility, such as thrombospondin-2 (*THBS2)* and proteolipid protein 2 (*PLP2*). *THBS2* is highly expressed in bovine ovarian small follicles than either medium or large follicles [52] as well as in small atretic follicles than healthy follicles [53].

Furthermore, the mitogen-activated protein kinases (MAPK), insulin, Wnt, gonadotropin releasing hormone (GnRH), and Notch pathways have also been identified, suggesting that some molecules of these pathways might also involved in sheep fertility or maintaining physiological activity of the ovary [54–56]. It has been suggested that oocyte quality, embryo development, and male fertility are affected by mitochondrial haplotypes [57–58]. Our data indicated that plenty of DEGs were linked to mitochondrial, such as glutaredoxin-2 mitochondrial glutamate carrier 1, and mitochondrial import inner membrane translocase subunit TIM44. In addition, proteins required for transport are also differentially expressed, such as zinc transporter 3, zinc transporter ZIP8 isoform 1, intraflagellar transport protein 57 homolog, and receptor-transporting protein 4. In summary, all of these results suggest that plenty of the DEGs are potentially critical for regulating sheep fecundity.

Previous studies demonstrated that growth differentiation factor 9 gene (*GDF9*), bone morphogenetic protein 15 gene (*BMP15*), and bone morphogenetic protein 1B receptor gene (*BMPR1B)* are the major genes affecting the hyper-prolificacy in sheep [59–61]. Similarly, a previous study showed that *BMPR-1B* gene and *GDF9* gene might be two candidate genes of fecundity in Qira black sheep [21]. However, the *BMP15*, *BMPR1B*, *GDF9* genes were not differentially expressed between Qira black sheep and Hetian sheep in this study. One potential reason for this finding is that there might be different genes present that change the gene expression patterns of *BMPR1B*, *BMP15*, *GDF9* and related genes or pathways affecting sheep fecundity. Another possible explanation is that there may be a result of using the whole ovary as material to determine differentially expressed genes. And these results are consistent with previous report on Small-tail Han sheep and Surabaya fur sheep ovaries using RNA-seq technology [20]. Anyway, our work will provide a valuable foundation for further molecular research of the sheep reproduction. Nonetheless, further studies are required to investigate the functional characterization of some selected DEGs, such as the expression profiling in ovarian follicles (or specific cell lines) at different cyclic stages in each breed, the functions in sheep follicular development, ovulation, hormone secretion and the regulatory networks.

## Conclusion

In this study, RNA-Seq technology was employed to analyze the DEGs in Qira black sheep and Hetian sheep in a genome-wide level. We found that 1,252 genes were significantly differentially expressed between the two libraries. KEGG pathway analysis indicated that some of these genes are involved in steroid biosynthesis, steroid hormone biosynthesis, TGFβ, Insulin, Wnt, Notch, and other signaling pathways. Our data indicated that there were many differences of ovary gene expression patterns between Qira black sheep and Hetian sheep. And the present study provides a very useful genetic resource that may contribute to a better understanding of the mechanisms involved in sheep litter size variations.

## Supporting Information

S1 Table Expression statistics for reference genes in the Qira black sheep and Hetian sheep. (XLS)

S2 Table Differential expression genes between Hetian sheep and Qira black sheep. (XLS)

S3 Table Significantly enriched pathway of differential expressed genes. (XLS)
